# Functions of patient- and family-centered pediatric cancer communication in Pakistan

**DOI:** 10.3389/fonc.2024.1393908

**Published:** 2024-09-11

**Authors:** Dylan E. Graetz, Alia Ahmad, Muhammad Rafie Raza, Ambreen Hameed, Asma Naheed, Atoofa Najmi, Afia tul Quanita, Shabnam Munir, Safwan Ahmad, Gia Ferrara, Courtney Staples, Carlos Rodriguez Galindo, Syed Ahmer Hamid, Sima Jeha, Jennifer W. Mack

**Affiliations:** ^1^ Department of Global Pediatric Medicine, St. Jude Children’s Research Hospital, Memphis, TN, United States; ^2^ University of Child Health Sciences, Children’s Hospital Lahore, Lahore, Pakistan; ^3^ Department of Oncology, Indus Hospital and Health Network, Karachi, Pakistan; ^4^ Department of Oncology, Dana Farber Cancer Institute/Boston Children’s Hospital, Boston, MA, United States

**Keywords:** pediatrics, cancer, communication, decision-making, global health

## Abstract

**Background:**

Communication is an essential aspect of high-quality patient- and family-centered care. A model for pediatric cancer communication developed in the United States defined eight communication functions. The purpose of this study was to explore the relevance of these functions in Pakistan as part of an effort to understand the role of culture in communication.

**Materials and methods:**

Semi-structured interviews were conducted with 20 clinicians and 18 caregivers of children with cancer at two major cancer centers. Interviews were conducted in Urdu or English and transcribed and translated as necessary. Two independent coders used *a priori* codes related to the communication model as well as novel codes derived inductively. Thematic analysis focused on operationalization of the functional communication model.

**Results:**

Clinicians and caregivers in Pakistan discussed the importance of all eight communication functions previously identified including: *information exchange, decision-making, managing uncertainty, enabling family self-management, responding to emotions, supporting hope, providing validation*, and *building relationships.* The operationalization of these functions was influenced by Pakistani cultural context. For example, *information-exchange* included the importance of addressing preconceptions and community myths, while *managing uncertainty* included strong references to religion and faith-based coping. Essential to all eight functions was *trust* between the family and the medical team.

**Discussion:**

These findings support the use of this functional communication model in diverse pediatric oncology settings and emphasize the importance of trust. Culturally sensitive operationalization of these functions could inform the adaptation of tools to measure communication and interventions aimed at supporting the needs of parents of children with cancer.

## Introduction

Communication is a central component of patient-centered pediatric cancer care. Families that experience “high-quality” communication have a heightened sense of purpose and peace of mind (1), are more hopeful (2), and describe increased trust of the medical team (3). Improved communication enables patients and caregivers to feel more comfortable caring for their disease at home (4) and aids in decision-making (5). On the other hand, patients and families who experience poor communication are at risk for decisional regret (6), emotional distress (7), and poor understanding (8).

In 2007, researchers at the National Cancer Institute in the United States proposed a model for communication that focused on interactions between adult cancer patients and their medical teams, identifying six interdependent communication functions: “building relationships”, “enabling family self-management”, “making decisions”, “managing uncertainty”, “responding to emotions”, and “exchanging information” (9). This model applies to communication tasks and outcomes from the time of diagnosis, throughout the cancer continuum and has been used for over a decade to define high-quality cancer communication including prediction of patients at risk for poor communication (10) and interventions aimed at improving cancer communication (11). In 2020, the model was adapted for pediatric cancer care and two additional functions, “supporting hope” and “providing validation”, were added (12). “Providing validation” includes ways in which clinicians empower parents, validate concerns, and assure them they are doing a good job. “Supporting hope” was operationalized as emphasizing positives, encouraging hope beyond cure, demonstrating intention to treat, and avoiding false hope, which might include, for example, avoiding the truth about a poor prognosis (12). Although the pediatric adaptation was published more recently, it has already been used in the United States to develop tools to assess pediatric cancer communication at the bedside (13).

While not always explicitly studied, all of the initial six functions have been noted in communication literature from low- and middle-income countries (LMICs) (14), where 90% of children with cancer live (15). Our prior work explicitly investigated these functions and found them to be prioritized by patients and families in Guatemala (16, 17). We have hypothesized that the equifinality of this functional model will make it adaptable for use in many low- and middle-income countries (18). Culture, including social norms, customs, religion, and traditions shape health-related beliefs and behaviors and are known to impact communication and decision-making during cancer care (19, 20). In order to further investigate how this model might apply or differ in distinct cultural settings, this study was conducted in two pediatric cancer centers in Pakistan. Pakistan is a lower-middle income country with a diverse population of approximately 200 million people who speak >75 different languages. Every year, an estimated 8,000-12,000 children develop cancer and <50% of these children survive (15, 21). Pakistan is a country with rich internal diversity as well as a population and healthcare system that is distinct from those in which this model has been previously applied. We conducted qualitative interviews with clinicians and caregivers of children with cancer treated at two pediatric cancer centers in Pakistan, exploring communication experiences and needs in order to examine the operationalization of this model in this unique setting.

## Materials and methods

The Consolidated Criteria for Reporting Qualitative Research Guidelines were used to ensure rigor in conducting and reporting this study (22).

### Settings and participants

We interviewed clinicians and caregivers at two of the nine centers caring for children with cancer in Pakistan: Indus Hospital and Health Network in Karachi and Children’s Hospital of Lahore. These hospitals are large referral centers. Each treat >1200 newly diagnosed pediatric patients a year, and both provide care to children free of charge. Clinician participants were eligible if they (1) spoke English, (2) were involved in the care of children with cancer, and (3) were employed at either center; purposive sampling was used to ensure a range of professionals. Caregiver interviews were conducted only at Indus Hospital and Health Network, as this team had the local human resources and training necessary to conduct qualitative interviews. Caregivers were eligible if they (1) had a child with cancer under the age of 19 years old who (2) had been diagnosed within the last 8 weeks.

We focused on diagnosis because it is a critical time for communication. It is a time filled with anxiety, fear, and uncertainty for families (23, 24) and is the medical team’s first opportunity to develop rapport and set the stage for an ongoing relationship as early treatment decisions are made. In addition, in Pakistan, like other LMICs, there is a high rate of treatment abandonment defined as the failure to start or complete curative therapy (25–27). High-quality communication from the time of diagnosis could minimize abandonment, a leading cause of mortality in these settings.

### Data collection

We conducted semi-structured interviews using a guide based on prior work (5, 28) and previously established functional communication framework (12, 18) (**Supplementary Figure 1**). Questions focused on experiences with communication at diagnosis including strengths, weaknesses, communication priorities, and unmet needs. Members of the research team (C.S. and G.F.) who did not work at either hospital conducted interviews with clinicians in English over Zoom. Interviews with families were conducted in person in Urdu by members of the research team (As N, At N, At Q, SM) in Pakistan. All interviewers had qualitative research training. Interviews lasted approximately 30-60 minutes, were audio-recorded, and professionally transcribed and translated into English as necessary.

### Data analysis

We analyzed transcripts using thematic analysis (29) beginning with an *a priori* functional model for communication previously described and highlighted for global pediatric oncology (12, 18, 30). Additionally, three authors (D.G., C.S., G.F.) iteratively read transcripts to identify novel codes based on recurrent themes. The codebook was developed through iterative review and application to 17 transcripts. Two researchers (C.S. and G.F.) independently applied codes using the final codebook (**Supplementary Table 1**) and MAXQDA software (VERBI, Berlin, Germany). Coders met to resolve discrepancies by consensus with a third-party adjudicator (D.G.) present as necessary.

## Results

We interviewed 20 multidisciplinary oncology clinicians, nine from Indus Hospital in Karachi, and 11 from Children’s Hospital Lahore. Clinicians included faculty, fellow, and non-faculty physicians, nurses, and psychosocial providers. We also interviewed 18 caregivers of children diagnosed with cancer at Indus including 11 females and 7 males (**Table 1**).

**Table 1:** Study participants.

Participants	Indus Hospital, Karachi		Children Hospital, Lahore		Total
	Female	Male	Female	Male	
Faculty Physician	3	1	4	–	8
Non-faculty Staff Physicians	1	–	3	–	4
Fellow Physicians	–	–	2	1	3
Nurses	–	2	1	–	3
Psychosocial providers	2	–	–	–	2
Parent	11	7			18
Total	17	10	10	1	38

Participants discussed all eight of the previously identified communication functions and highlighted the importance of “trust” as an element underpinning each function.

### Information exchange

All participants discussed “information exchange.” Clinicians and caregivers emphasized that information exchange should be bidirectional and discussed the importance of addressing cancer preconceptions (**Table 2**). Successful communication, according to participants, depended on families’ ultimate understanding of their child’s diagnosis, prognosis, and treatment plan. One clinician described this saying: “The initial or the general impression that most parents have [is that cancer is] just like a death [sentence]. And when we try to explain to them that this is a curable disease and the prognosis is not as bad and they can be cured and they can live a normal routine life … then they do understand and it helps” (Faculty Physician, Male, Indus). Many caregivers and clinicians also discussed the importance of sharing information with the pediatric patient, particularly adolescents.

**Table 2:** Manifestation of each communication function.

Communication Function	Manifestation as perceived by providers	Manifestation as perceived by parents
Information Exchange	Bidirectional information exchange “I think, good communication not only helps you– not only does it help the parents and the patient understand what you’re doing, but many times it also helps to let us know what we’re doing, how we see, and if there are any issues that the family is facing or the patient is facing. Somethings, not all things are recordable by monitors and laboratory people findings. This helps many a times the patients speak to us or the parents tell us things and that’s only when we have good communication with them.” (Faculty Physician, Male, Indus) Addressing preconceptions “So the solution for this is only the better communication because there are many like these all problems of social stigma for these myths, what we need is communication because we’re giving them education.” (Faculty Physician, Female, Lahore) Establishing understanding “Most of the times they do understand about when we tell them about the treatment overview, that how this disease – how much does this disease curable and what is the prognosis, and what will be the treatment course, whether it include radiation or chemotherapy, they do understand about it.” (Non-faculty Staff Physician, Female, Indus) Sharing information with the child “We usually talk to them and explain the diagnosis to them, if they’re old enough to understand. Especially, what I’ve seen is that the teenage patients are very much interested to know about their disease.” (Non-faculty Staff Physician, Female, Indus)	Bidirectional information exchange “He asked me if I knew what disease she had. I told him that I knew. He looked at me and then asked what disease she had. Then I told him that she had cancer in her blood. … He asked if I was worried. I said of course I was worried since I only have one daughter. He then just told us that this [bruise] that she has, pay attention to it and that if it gets bigger, we should tell the doctors. He also said that if blood comes out from anywhere, her eyes, nose, mouth, nails, [or from her bruise] or is in her urine, we should immediately inform the doctors.” (Mother, female patient, Indus) Addressing preconceptions “I used to think that this has no treatment. But now I think that this has treatment and when children come to Indus, they let them leave only when they are cured. Now I am very reassured.” (Mother, female patient, Indus) Establishing understanding “She gave me all the information. I had an interview with her and me and [patient] understood it all with ease and we left here very relaxed.” (Mother, male patient, Indus) Involvement of the child “She also found out about her condition when she came here. I tried to hide it from her. Doctors don’t really hide anything, they say everything in front of the patient.” (Mother, female patient, Indus)
Making Decisions	Including family in decision-making “Yeah, in the first conversation, in the initial days of the treatment – when you’re going to decide the treatment plan, when you make the treatment plan, you do want the parent to be on board then, so that they know what is going to happen.” (Non-faculty Staff Physician, Female, Lahore) Discussing risks/benefits/side effects “My role is that after the initial diagnosis, we [explain] the course of disease and the most likely outcome the prognosis, the side effect of the chemotherapy, and after taking the consent explaining every [thing] then we start the treatment.” (Fellow Physician, Male, Lahore) Supported decision-making “We don’t generally stop them or advise them against the spiritual healer because of the fact that it is such a strong belief and we don’t want them to quit treatment as a result of us saying that or advising against it. So, we just advise them to continue treatment here as well as going to the spiritual healer because the spiritual healer does not generally give them medication right. So, as long as it’s not hampering our or making them miss their appointment dates at the hospital while they’re getting treated. We don’t have an issue with that” (Psychosocial Provider, Female, Indus)	Including family in decision-making “We usually talk to the elders and they make the decision and in regards to decisions related to a disease, I spoke with my husband … In my house, I live with my mother-in-law and husband and whatever decision they make, I accept it.” (Mother, male patient, Indus) Discussing risks/benefits/side effects “The doctor said they will start the medicine. Due to the medication, my child might face some problems but [medication] needs to be administered. But we will come for our child’s sake no matter what we have to do. For my child’s sake, I have to get the treatment done for my child.” (Mother, male patient, Indus) Competing priorities “My husband does not have much of an income. We live in a rented house. So you yourself can imagine. His total salary is 28,000 rupees … There are three children … when [our child was diagnosed] we had heard that the treatment for this is very expensive … Your two children or three children, how many there are and whoever the family members are, you need to give priority to this [sick] child. To his eating and drinking, or whatever it is, meaning bathing and cleaning, giving medications on time, you have to do all this only” (Mother, male patient, Indus)
Managing Uncertainty	Exploring unknowns and answering questions “What I’ve seen in my personal experience is that kids or children when they’re coming into the hospital they’re surrounding by a lot of ambiguity … one of the key things I always like to tell the families that I come into contact with is that let’s take it one step at a time” (Psychosocial Provider, Female, Indus) Providing reassurance “Helping them feel grounded at that point in time that you know where at one point here let’s deal with this and then we’ll deal with the next thing, and we’ll be with you.” (Psychosocial Provider, Female, Indus) Overcoming denial “Mostly, the families react with a denial phase … they don’t accept the diagnosis of the cancer. And in these cases, we have to give an extra time for counseling … sometimes they will need counseling again and again to remove their denial phase, and to accept that this is the disease and it is curative, and the patient has to be treated.” (Faculty Physician, Female, Indus) Role of religion “From religious point of view, that helps us a lot to put them at peace, that this is something from our God, and we have to accept it. And we have to keep our strong faith on Allah, that whatever he has decided for us is the best.” (Faculty Physician, Female, Lahore)	Exploring unknowns and answering questions “I asked about so many children suffering from cancer. Why? So this answer came that the inventors of chemo and medication, even they do not know.” (Mother, male patient, Indus) Providing reassurance “Doctor helped us a lot. He explained to us in such a way, very lovingly, that we became prepared for this that we have to get this test done. That really, our daughter will get better … And you would not believe it but Doctor gave her clothes, toys…”(Mother, female patient, Indus) Overcoming denial “They conveyed it to me over there too but I did not understand it. I thought that [they] were lying and that my daughter only has a fever.” (Mother, female patient, Indus) Role of religion “This child is given by God and if He wants to take it back, then He will and only He can give me patience to bear it. And if God will give it then He is the one to grant me happiness. I am not hopeless with my God.” (Mother, male patient, Indus)
Enabling Family Self-Management	Caring for the child at home “It’s not just that they need to come to the next appointment … it’s basically the caretaker at home, needs to understand well, how to take care of that cancer child at home as well. And what are the danger signs when they need to bring the child to the hospital – back to the hospital.” (Non-faculty Staff Physician, Female, Indus) Anticipatory guidance “We have a few pamphlets displayed in our corridors regarding infection control, regarding febrile neutropenia, regarding side effects of chemotherapy. With a few pictures, so if the parents are not literate, they can get some ideas from the pictures too.” (Nurse, Female, Lahore)	Caring for the child at home “They said to look after her cleanliness, to give her medications of set times in the morning, and to change her clothes in the morning, bathe her, and to feed her good food. To not feed her anything with a skin.” (Mother, female patient, Indus) Anticipatory guidance “When he went through his first chemo, he suffered from fever. So the doctor [name redacted] had already told us that when he goes through his chemo, he will get a fever. Whenever the fever comes, whether it is day or night, we are supposed to come to emergency” (Mother, male patient, Indus)
Building Relationships	Overcoming hierarchy “In Pakistan the culture usually revolves around being scared slightly after healthcare practitioners … so parents and anybody that comes into contact with them don’t generally communicate their concerns or ask too many questions.” (Psychosocial Provider, Female, Indus) Demonstrating care/concern “They understand that doctors are important. They know inherently that this is what they’re here for, but here we need to constantly with every new patient coming in, we have to build that understanding for them that this is what we’re here for and you can come to us with your queries or any conversation that you need to have whether difficult for you or easy for you.” (Psychosocial Provider, Female, Indus) Availability “Most of us have given our own phone numbers that even if you have a problem, you can contact us personally … even out of the hospital hours, if they have a problem, we are mostly, happy to help them.” (Faculty Physician, Female, Lahore) Provider consistency “When they have the first encounter with us they don’t trust us, but later on enough time when they have built up a relationship with us after one month or after a few weeks they start trusting their own physician and then they try to … stick to one consultant and one physician always. I have a few patients that who always come to my [clinic] and whichever course they are on our whichever complication they’re having, they come to my [clinic].” (Faculty Physician, Female, Lahore)	Overcoming hierarchy “Doctor is a very good person He did not make us feel how a doctor usually does. It was as if he was a member of our family. With love, he explained to my husband and me … and we understood.” (Mother, female patient, Indus) Demonstrating care/concern “When the doctors would comfort, meaning comfort is a big support of a human being. It is the support of one another. Just this the strength the doctors etc. give. That gives strength.” (Mother, female patient, Indus) Guidance “Always stay in touch with the staff, doctors. They will help you a lot and act like a guide with you so getting the treatment done will become easy for you.” (Mother, male patient, Indus) Gratitude “When we came here, the staff consoled us and the doctor, I am really grateful of him because he, I do not know what to say … there are no words … he gave me much strength, comforted me and consoled me, my husband and my sister too.” (Mother, male patient, Indus)
Responding to Emotions	Addressing fear and concerns “I think that once the health care providers communicate [emphatically] with the parents and patients it helps much more … When we respond to their emotions, their fear, their anxiety, their concerns and then we answer their concerns, their anxiety, then they build the trust on us, then it all the, these things make the communication easy … When we solve the issues concern which are going in the mind of the patient and the patient parents is their fear, their anxiety, then we resolve, then we answer them this thing then they if they this trust make us strong as a provide care provider.” (Faculty Physician, Female, Indus) Holding space for emotional responses “We have psychologists who do proper psychotherapy to make sure that they’re processing how they’re feeling, and they have a very good understanding and like, and they’re dealing with their emotions, so that really helps” (Nurse, Male, Indus)	Addressing fears and concerns “In the beginning I was worried about my child and what will happen to her and will she ever get better. Then when I met with one of the staff members of this hospital … she explained to me and made me sit with her and consoled me … I received much comfort after listening to her” (Mother, female patient, Indus) Lack of emotional support “No one gives us emotional support. Our hearts cannot tolerate it anymore. This pain is unbearable for us. He is just a child. I am a mother. *sobbing* No one knows what is going through my heart. I will do anything … just to see my child recover. His recovery is everything for me.” (Mother, male patient, Indus)
Supporting Hope	Emphasizing the positives “We try to explain to them that this is a curable disease and the prognosis is not [so] bad and they can be cured and they can live a normal routine life.” (Faculty Physician, Male, Indus) Demonstrating intent to treat/cure “Most of the population usually think like this that cancer is untreatable, and it’s life threatening. But after our initial counseling … they are satisfied, treatment is there and it can get cured by team work and with their help also.” (Fellow Physician, Male, Lahore) Avoiding false hope “We cannot give him false hope. We have to tell him like a friend that this will not cure the disease, this will only improve his or her symptoms.” (Faculty Physician, Female, Indus)	Emphasizing the positives “Then a doctor [counselor] over here said we will tell her about it. Then (she) explained to her about the disease and that … there will be stressors but she will get better.” (Father, female patient, Indus) Demonstrating intent to treat/cure “When I came here, doctors told me that his cancer is not that much and will be able to get treated … He will be fine. There is a treatment for him here.” (Mother, male patient, Indus) Addressing misconceptions “I got hope that my child will get better. Before, this hope was not there because in the area we live in, cancer is associated with death. Whoever has cancer is assumed to die.” (Mother, female patient, Indus) Utilizing survivor stories “The pictures they showed of the children who were little and they have grown up now, I found hope, and how they got properly treated and now they have aged up.” (Mother, female patient, Indus) Religion/Faith “I have a lot of hope in God, He will end it God willing.” (Mother, male patient, Indus)
Providing Validation	Reinforcing good parenting beliefs “I and my other colleagues, we try to give them this assurance that your kid is just fine. He has just caught a disease, which is like any other disease. And the best part is that you [came] to the institution where the treatment is being offered.” (Non-faculty Staff Physician, Female, Lahore) Engendering solidarity “When the patient came in the hospital … we empathize with the family. We said, okay, we know it’s a very difficult time for you and for the kids, for the whole family, but you are not alone in this time, in this day, in this whole journey, we are with you.” (Psychosocial Provider, Female, Indus) Validating concerns “Validating their experiences, validating their emotions regarding all those fears.” (Psychosocial Provider, Female, Indus)	Reinforcing good parenting beliefs “We did not think that our decision was wrong one. Our decision remained good and everyone tells me that I have made a good decision about Indus.” (Mother, male patient, Indus) Alleviating blame “We thought that it was our fault somehow for the condition of our daughter but the doctor said that was not the case.” (Father, female patient, Indus)
Trust	Overcoming distrust/misinformation “In past years doctors were so respected in our culture and in our community, but with the passage of time … it has a little bit deteriorated. I don’t know what’s the reason, but it has. So maybe … they have taken a lot of information from the social media and from other internet and all that … so there is a kind of mistrust” (Faculty Physician, Female, Lahore) Competence based trust “At that time, the first visit … Much of the family is not understanding, but they say that then if you are saying [this] as a physician, that [the] disease is preventable … maybe [we] can trust you” (Faculty Physician, Female, Indus) Relationship based trust “There’s rapport between the doctor and the parents, right, and that relationship develops they have a strong faith on the team that what they’re doing is good and they do not worry anything that what is going on.” (Faculty Physician, Female, Lahore)	Overcoming distrust “I was terrified of the word Cancer. I did not believe the doctor and thought they were lying when she said my daughter had Cancer. My daughter was always very healthy.” (Mother, female patient, Indus) Competence based trust “Whatever opinions the doctors give we do exactly that.” (Mother, female patient, Indus) Relationship based trust “We gained a lot of information, information from everyone and here at Indus … my experience and other people’s experience has been of really good people with whom we have good connections, who we trust and who give us confidence.” (Mother, male patient, Indus)

### Decision-making

Every interview included “decision-making.” Participants discussed the importance of involving the family in decision-making, including extended family members. Clinicians and caregivers also identified a discussion of risks, benefits, and side effects as important to the decision-making process (**Table 2**). Caregivers explained their competing priorities and how factors beyond the cancer center including geography, family income, and other children weighed on them as they made decisions. Caregivers and clinicians described how acknowledgment of these competing factors during diagnostic communication helped families accept care. One caregiver said, “The [psychologist] helped us a lot … she asked what happened, how it happened and I told her about my house’s situation and how our life is going like this and said that really, you have seen many worries and when a child gets sick, the entire house gets affected … [she] said that don’t worry, her test will be done in the same way as other kids. But she has a little problem with her brain, hence we would need brain doctors … As soon as the doctor called us, we went” (Mother, female patient, Indus). Clinicians also emphasized the importance of supported decision-making, including recognizing traditional beliefs and allowing families to continue traditional therapies as long as they did not interfere with allopathic care.

### Managing uncertainty

“Managing uncertainty” was identified in every interview. Components of this function included exploring unknowns and answering questions, providing reassurance, overcoming denial, and the role of religion (**Table 2**). One clinician described how she handles persistent uncertainty by acknowledging the unknown and providing small amounts of information at a time: “So we will tell them that your child now this is happening now … we will go there, so they absorb easily. If we tell all the things [at once] I think – for them [it causes] too much worry … In every disease we can’t predict all the things in the first go. And we can’t predict, we don’t know [how the child will react once] we give the chemotherapy … We can’t give them 100% that your child will survive” (Fellow Physician, Female, Lahore). Caregivers and clinicians both discussed the solace they found in religion and the role of God, or Allah, and how they used this shared faith to help manage uncertainty. One parent said: “I believe in God. We have come this far and the surgery will also happen God willingly and God will take care of it” (Mother, female patient, Indus).

### Enabling family self-management

Every interview referenced “enabling family self-management” which manifested as caring for the child at home and anticipatory guidance regarding what to expect and when to bring the child back to the cancer center (**Table 2**). Caregivers and clinicians discussed the role of effective communication in not only making sure the patient made it to their appointments, but also that the child was well cared for at home, including following specific guidance regarding hygiene and nutrition. Taking care of a child with cancer was a constant responsibility. As one parent described: “For my child, I am always ready. Day or night for [my child] for 24 hours I am ready to give him attention and put my other tasks to one side. All my time is for [my child]. [My child] needs to eat this now, [my child] needs to get up, [my child] needs to sleep, [my child] needs to take a bath, [my child] needs to study so it is all of my effort to give all of my attention to [my child] and whatever advice the doctors gives or you give, in accordance with that I keep my child prepared at home like that” (Mother, male patient, Indus).

### Building relationships

“Building relationships” was identified in every interview. Caregivers and clinicians referenced a cultural hierarchy separating physicians from patients that had to be overcome at the start of a therapeutic relationship and explained how clinicians established rapport by demonstrating care and concern (**Table 2**). As one parent said: “Doctor X appreciates a lot and loves my child very much … she lets me know about everything … I am very at ease with her and the staff” (Mother, male patient, Indus). Clinicians further described how they built relationships by making themselves available to families and providing a consistent presence. Over time, clinicians explained, families began to trust and seek out specific clinicians. As caregivers described this relationship, they emphasized the guidance available clinicians provided them and the gratitude they felt toward the medical team: “It is their kindness that they will get the treatment done … I am trying to thank them. Aside from this, I have nothing to offer them. I can only thank them and pray for them” (Mother, female patient, Indus).

### Responding to emotions

All clinician interviews and 17/18 caregiver interviews included “responding to emotions.” The dominant emotions discussed were anxiety, fear, worry, and concern. This function manifested as clinicians addressing and responding to those fears and concerns. As one parent said: “[When we come here (initially), we are all very worried]. Believe me that our worry was so much when we were coming here but just a little bit of [clinician] attention, the doctor’s attention, or a social department’s attention is a source of much happiness for us. And I become relaxed” (Mother, male patient, Indus). Clinicians specifically discussed how a multidisciplinary team including psychologists allowed them to hold space for emotional responses to treatment. On the other hand, a few parents described unmet emotional needs and a lack of emotional support (**Table 2**).

### Supporting hope

“Supporting hope” appeared in most (16/20) clinician interviews and all but one caregiver interview (17/18). In both clinician and caregiver interviews, supporting hope manifested as emphasizing the positives and demonstrating intent to treat or cure (**Table 2**). Clinicians discussed the importance of avoiding false hope, especially in the setting of poor prognosis. Caregivers described how clinicians addressed misconceptions and utilized survivor stories to further support hope. Caregivers also frequently discussed how their faith gave them hope, and how clinician’s references to God supported this hope. At times, caregivers connected God (Allah) to the medical team: “I am … very hopeful in God and the doctors too” (Mother, male patient, Indus).

### Providing validation

“Providing validation” was identified in half (10/20) of the clinician interviews and some (7/18) caregiver interviews. This manifested as reinforcing good parenting beliefs and minimizing regret, particularly around decision-making. As one parent described “[My child’s clinician] said okay, you have made such a good decision” (Mother, male patient, Indus). Clinicians further described how they empathized, validated caregiver concerns, and partnered with families to engender solidarity. For caregivers, providing validation also manifested as alleviating blame or guilt (**Table 2**).

### Trust

In previous models, trust has been explicitly discussed as a component of “building relationships” and described as an outcome of high-quality communication. In this study, participants described “trust” existing before and throughout diagnostic communication. “Trust” was identified in 75% (15/20) of clinician interviews and about half of the caregiver interviews (8/18). Participants described overcoming initial mistrust stemming from disinformation and depicted both competence-based trust as well as relationship-based trust (**Table 2**). Competence-based trust included a deference to clinicians based on their position or title and expected knowledge. One parent described this as close to God “after God, doctors are responsible for making this treatment successful and curing this disease” (Mother, male patient, Indus). Relationship-based trust, on the other hand, stemmed from rapport that was built over time: “Every time [there is] good communication … patient and parents increase [their] acceptance of the treatment … so the treatment compliances increases and their … understanding is increasing their trust … and their false misconceptions decreasing, their distress is decreasing and … their trust in their physician increases” (Faculty Physician, Female, Indus).

## Discussion

This multicenter study demonstrates that a functional model for pediatric cancer communication developed and previously applied in high-income western settings (12) is relevant in Pakistan, a lower-middle income country located in the Middle East. Our findings confirm prior work exploring the possibility of applying this model to diverse settings with variable resources (18).

The specific manifestations of these functions, and the extent to which they were discussed by Pakistani participants highlight the importance of culture and the equifinality of the model. All eight functions were discussed by a majority of both clinician and parent participants, with 5/8 functions (information exchange, decision-making, managing uncertainty, enabling self-management and building relationships) identified in every interview. Supporting hope was discussed by a higher percentage of parents than caregivers, while providing validation and trust were discussed in more clinician interviews. Hope has been a major area of focus in communication research in pediatric oncology both from high-income (31) and low-income contexts (17). Our data confirms the importance of honesty highlighted in prior literature and emphasizes a connection to faith or religion as helpful to supporting hope within the Pakistani context. The role of religion was also discussed by clinician and caregiver participants in relation to managing uncertainty. Pakistan is a predominantly Muslim country and this shared faith emerged as important in diagnostic conversations in a way that has not been described in similar studies conducted in the United States.

Information exchange and decision-making have also been the focus of communication work in high-income countries and were prioritized in work from Guatemala (16), another middle-income country that is geographically and culturally distinct from Pakistan. While this may indicate the relative importance of these two functions in all settings, it is also possible that there are specific conditions in LMICs that contribute to their emphasis by participants. For example, limited childhood cancer awareness, misconceptions, and low health literacy may contribute to the need for explicit and thorough information exchange in LMICs. Similarly, decision-making in these settings may be complicated by extended family involvement and traditional beliefs. It is also possible that different communication functions emerge as more or less relevant at different times throughout the cancer care continuum. This study, as well as those conducted in Guatemala (5, 16), focused on diagnostic communication, a time during which there is a lot of essential information that must be exchanged, and many decisions must be made.

**Figure 1:**
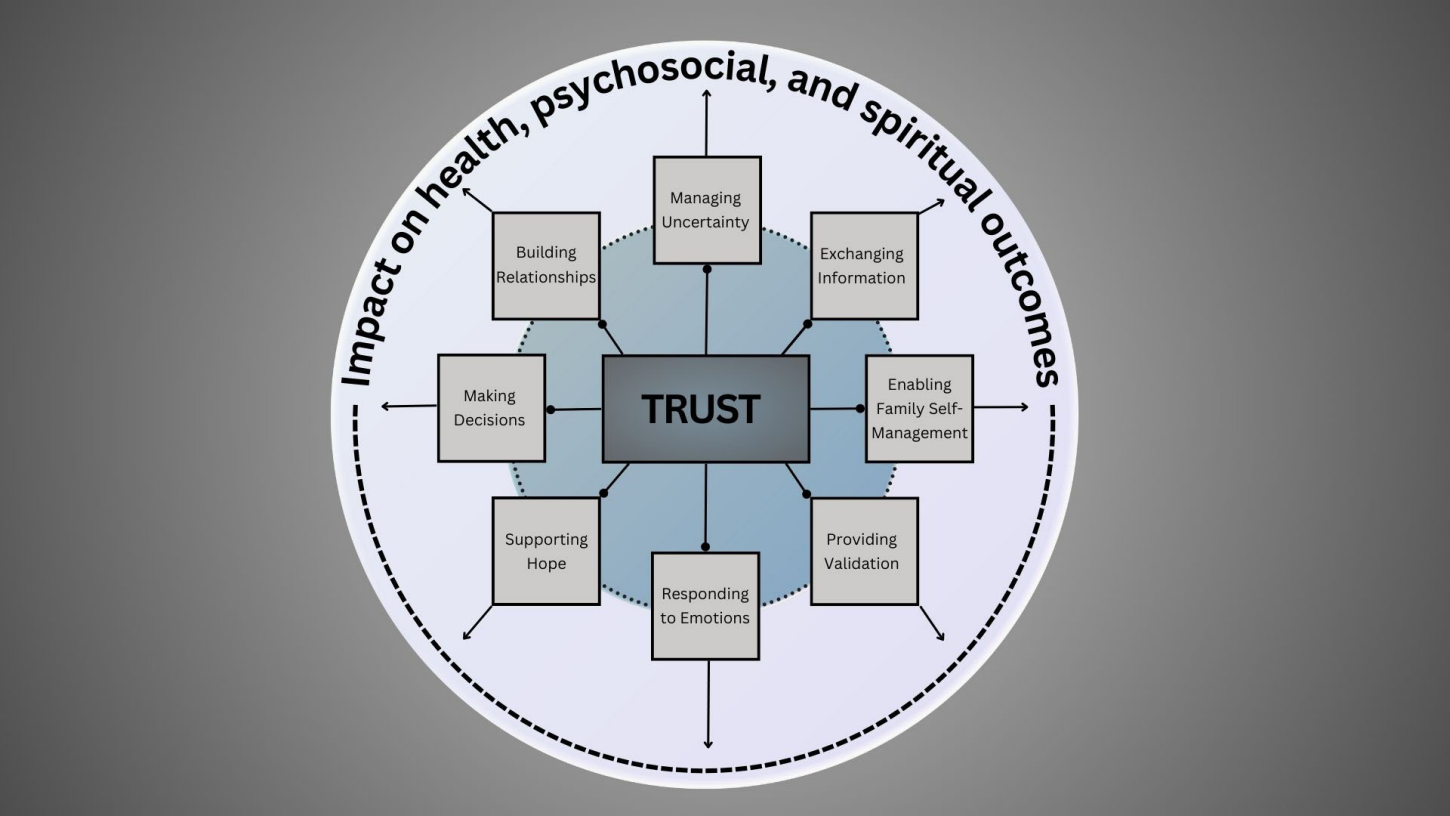
Model for patient-centered communication in Pakistan.

In addition to the eight identified communication functions, participants in this study discussed the importance of trust. Previous models implicitly mention trust as a component of many functions and specifically incorporated trust as a component of “building relationships,” while also highlighting it as an outcome of high-quality communication (9, 12). Clinicians and caregivers in this study discussed the importance of trust before and during diagnostic communication. Many acknowledged that families had to overcome distrust in the medical system before accepting care and others described competence-based trust (32, 33) that was related to a family’s prior experiences or inherent faith in the medical team rather than the actions or words of the clinician. Over time, relationship-based trust (32) was established. Actions taken by the medical team and time spent caring for a child with cancer led caregivers to overcome their initial distrust or built upon the competence-based trust initially established. Trust, as described by participants in this study, was a separate and explicit function of communication. We have placed it at the center of our adapted model because participants describe trust as necessary to the effectiveness of all other functions of communication, which in turn impact outcomes (**Figure 1**). It is possible that the importance of trust is specific to the Pakistani context or other settings in which initial mistrust could contribute to delayed diagnosis and abandonment of therapy. However, competence- and relationship-based trust have been previously described in literature from high-income countries and we hypothesize that this modified model, in which trust is unique and central to all other interrelated functions, might be applicable in other pediatric cancer settings. Further work exploring this hypothesis is warranted.

An assessment tool to measure these communication functions as experienced by caregivers and pediatric patients (PedCOM) has been previously developed and validated in the United States (13). This tool includes questions based on each function, some of which we have previously adapted and utilized during studies in Guatemala (5, 16). Given the relevance of this model to Pakistani populations, the tool and question items should be translated and adapted for further investigations of patient-centered communication in this setting.

Additionally, process models and interventions are needed to operationalize this functional model and apply it to improve care for children with cancer in Pakistan and around the world. There are very few communication interventions for children with cancer (34), and to our knowledge none that have been studied in Pakistan. Findings from this study might be used to inform process models and specific interventions aimed at improving communication and tailored to the Pakistani context.

This in-depth study explored communication functions in Pakistan, a setting where they have not been previously explored, using a model from existing literature. However, certain limitations should be considered as our findings are interpreted. We interviewed clinicians at 2 centers treating children with cancer in Pakistan and caregivers at only 1 site. While we believe the themes discussed are applicable to all centers in Pakistan and potentially pediatric cancer communication in other LMICs, further context specific work is needed. Additionally, all interviews were conducted around the time of diagnosis. Future work should explore how this model or specific communication functions might be perceived throughout the cancer care continuum. Finally, all data analysis was conducted in English which may have impacted our ability to interpret nuanced results.

## Conclusion

Communication is essential to high-quality pediatric cancer care in low- and middle-income countries where it may improve trust in the medical team, enable parents to feel more comfortable making decisions and caring for their child at home, and decrease treatment abandonment. Our findings support the use of a functional model for communication in Pakistan and emphasize the importance of trust. Adaptation of existing measurement tools should be used to further investigate this model and culturally sensitive operationalization of all communication functions should influence interventions aimed at supporting the needs of caregivers and children with cancer in diverse settings.

## Data Availability

The raw data supporting the conclusions of this article will be made available by the authors, without undue reservation.
