# Supplementary material for: Functions of patient- and family-centered pediatric cancer communication in Pakistan
Source: Front Oncol. 2024 Sep 11;14:1393908. doi: 10.3389/fonc.2024.1393908 (PMC11422343; doi:10.3389/fonc.2024.1393908)
Supplement: Supplementary file 1 [file Table1.docx]

**Supplemental Materials**

Functions of Patient- and Family-Centered Pediatric Cancer Communication in Pakistan

Dylan E. Graetz MD MPH ^a^, Alia Ahmad MD^b^, Muhammad Rafie Raza MD^c^, Ambreen Hameed MD^b^, Asma Naheed MSc^c^, Atoofa Najmi MSc^c^, Afia tul Quanita BS^c^, Shabnam Munir BS^c^, Safwan Ahmad MBBS^b^, Gia Ferrara MSGH ^a^, Courtney Staples MS^a^, Carlos Rodriguez Galindo MD ^a^, Syed Ahmer Hamid MD^c,^ Sima Jeha MD ^a^, Jennifer W. Mack MD MPH ^d^

^a^ St. Jude Children’s Research Hospital, Memphis, Tennessee, USA, ^b^ University of Child Health Sciences, Children’s Hospital Lahore, Lahore, Pakistan, ^c^ Indus Hospital & Health Network, Karachi, Pakistan, ^d^ Dana Farber Cancer Institute/Boston Children’s Hospital, Boston, Massachusetts, USA

**Table of Contents**

| **Supplemental Material** | **Page** |
| --- | --- |
| Supplemental Figure 1: Interview guide | 2 |
| Supplemental Table 1: Codebook | 3 |

**Supplemental Figure 1: Interview guide**

**Interview guide for key informants (providers)**

*Thank you for agreeing to participate in the parent and provider communication project.*

*We appreciate your time and we are grateful that you are sharing your experience and insight with us.*

- *To begin we will review the purpose of this study and main points of the informed consent,*
  - *This purpose of this study is to learn about the processes for diagnostic communication at [Indus or Children’s Hospital Lahore]. We are hoping to hear your honest opinions and reflections.*
  - *This interview will remain confidential and those you work with will not have access to the topics we discuss.*
  - *As a reminder, this interview will be recorded*
  - *We will take measures to protect the privacy and security of all your personal information, but we cannot guarantee complete confidentiality of study data*
  - *Taking part in this research study is voluntary.* ***You have the right to choose not to participate.*** *You can stop being in the research study at any time*
- *To promote optimum communication, interviews should be conducted with high-speed internet connection which will allow for uninterrupted audio and video.*
  - *May take a moment here to ask about internet connectivity*
- *Should connection issues occur, please utilize the chat box in the bottom right corner of the WebEx window or contact the interviewer through WhatsApp or email if the chat is not functioning. Video can be disconnected if connection interruptions continue.*
  - *Interviewer can put contact information (email address/Whatsapp) in the chat*
- *At any point, please ask the interviewer to repeat a question, if needed and for any reason.*
- *If an interviewee needs to stop the interview or the connection ends abruptly, please contact the interviewer by email or whatsapp to reschedule.*

*Ascertain that the interviewee is in a good, private place to begin the interview. If so, begin*

1. Can you explain to me your role in the hospital?
   1. What is your role in communication with newly diagnosed parents/families?
2. Would you please describe to me the process for newly diagnosed cancer patients at your hospital?
   1. How do they learn about the diagnosis? Who tells them?
   2. Where do these conversations happen?
   3. How much time do these conversations take?
   4. Who else is part of these conversations? Are extended family members included? Are other members of your team present?
   5. How do you feel these conversations go?
   6. What are the pieces of diagnosis/treatment planning that families seem to understand well?
   7. What things are harder for families to understand?
3. Can you tell me a little bit about your patient population?
   1. What are the cultural backgrounds of your patients?
   2. Are there traditional or religious beliefs that affect how families feel about cancer? Or treatment?
   3. What language(s) do most of your patients/families speak?
      1. Does this affect communication with the team?
   4. What is the education level of most of your patient families?
   5. What do you think most of the parents you see understand about cancer before their child is diagnosed?
      1. Where have they learned these things?
4. Do you think communication is an important part of care for children with cancer?
   1. Why or why not?
   2. Do you think it has an impact on outcomes?(potential probes re: survival, abandonment)
5. What are your center’s biggest strengths in terms of communication with families?
6. Can you give me an example of a time you were involved in communicating a new diagnosis and treatment plan with a family and communication went well?
   1. What made it go well?
   2. Were there things you (or your team) did that you think were helpful?
   3. Were there things about this family that made it easier to talk to them?
   4. How do you think this communication helped the family?
   5. Did this communication affect the family’s relationship with the care team moving forward?
7. What are your biggest challenges in communication of new diagnoses and treatment plans?
8. Can you give me an example of a time when communication did not go well?
   1. What made talking to this family difficult?
   2. How did this affect the family’s relationship with the care team going forward?
9. If you could change one thing or make one aspect of communication easier, what would it be?
10. Is there anything else that you think it is important for us to know about communication at [Indus or Children’s Hospital Lahore]? Any concepts we did not cover?

**Interview guide for key informants (parents)**

*Thank you for agreeing to participate in the parent and provider communication project.*

*We appreciate your time and we are grateful that you are sharing your experience and insight with us.*

- *To begin we will review the purpose of this study and main points of the informed consent,*
  - *This purpose of this study is to learn about the processes for diagnostic communication at [Indus or Children’s Hospital Lahore]. We are hoping to hear your honest opinions and reflections.*
  - *This interview will remain confidential and your medical team will not have access to the topics we discuss or the information you provide.*
  - *As a reminder, this interview will be recorded*
  - *We will take measures to protect the privacy and security of all your personal information, but we cannot guarantee complete confidentiality of study data*
  - *Taking part in this research study is voluntary.* ***You have the right to choose not to participate.*** *You can stop being in the research study at any time*
- *At any point, please ask the interviewer to repeat a question, if needed and for any reason.*

1. Tell me about your experience coming to Indus…
   1. How did you know about this hospital?
   2. What made you come in?
   3. What happened once you got here?
2. Before your child was diagnosed with cancer, what did you understand about the disease?
   1. Had you heard the word cancer before? What had you heard? Who had you heard this from?
      1. How did you learn about cancer? Where did you look for information?
   2. What do people in your community think about cancer?
3. Once you got to Indus, who explained cancer to you?
   1. How did they explain it to you?
      1. Where were you when they told you?
      2. Who was with you?
      3. What was the process for communication?
   2. How was that similar/different to what you already understood or believed about cancer?
4. What do you understand now about your child’s diagnosis?
   1. How did you reach this understanding?
   2. Is this similar to what your family/community thinks about your child?
   3. Do you still have questions or concerns?
5. There are lots of decisions a family has to make, for example some families have to make decisions about how to spend money, or whether to send their children to school. How do you usually make decisions in your family?
   1. Who is responsible for making decisions?
   2. Are you involved in decision making? How?
6. I imagine you have had to make many decisions here at Indus. How has making these types of decisions been similar or different to other decisions your family has had to make?
   1. What has helped you make decisions about your child’s diagnosis/treatment?
   2. Is your child involved in these decisions?
   3. Have there been any disagreements within your family related to your child’s cancer care?
7. Often doctors, nurses, and psychologists help talk to parents about their child’s diagnosis and cancer treatment. How has the team here at Indus talked to you about your child?
   1. Who has been most helpful in explaining your child’s cancer and its treatment? How did they explain things?
   2. What did the team say that you thought was most important or helpful for you in understanding your child’s diagnosis and treatment?
   3. Do you feel supported emotionally by the team? If yes, how have they shown their support? If no, how could the team better support you?
   4. How has the team helped you make decisions about your child?
   5. What has the team told you about how to take care of your child at home?
      1. Do you feel prepared to take care of your child at home?
      2. Do you feel you will be able to do what is being asked of you?
   6. Are there things you are still wondering about or do not understand about your child’s cancer?
8. If you had the opportunity now to speak with other parents of a child recently diagnosed with cancer, what would you tell them about talking to the doctors and nurses here? What advice would you give them?

**Supplemental Table 1: Codebook**

| **Code** | **Definition** |
| --- | --- |
| Information exchange | Parents seek information about the cause, diagnosis, treatment, prognosis, and lasting effects of cancer and its treatment. Fulfilling information needs not only helps families to gain important knowledge about a child's illness but also aids the development of a strong clinician-family relationship and supports decision-making, among other outcomes. Patients and families also have information that they want to share with clinicians, so exchanging information seeks a bidirectional understanding between clinicians and families. This code should be used for all information exchange between a patient/family and the medical team, even if it is not bidirectional in nature. |
| Making decisions | Effective decision-making requires effective communication. Such communication can support decision-making in a number of ways: raising the clinician's awareness of the family's needs, values, and fears; clarifying clinical reasoning and treatment options; and alerting the clinician to the family's preferred role in decision-making. At other times, decisions might be presented by the clinician as strong recommendations. |
| Managing uncertainty | Parents and patients experience many types of uncertainty after a diagnosis of cancer. This uncertainty can pertain to prognosis, side effects, frequency of hospitalization, and long-term effects, among others. Family-centered communication should help to communicate clinical uncertainty when it exists, dispel uncertainty when there is an answer, and help parents and families manage unavoidable uncertainties. Parents and patients also identify uncertainties about possible future outcomes and communicate bidirectionally with clinicians to obtain anticipatory guidance for these uncertainties. |
| Enabling family self-management | Parents must manage complex medical, logistic, and emotional challenges within their families. Communication that enables parents to address these ongoing challenges can support family self-management. Example: when the providers review the guidelines that should be followed for treatment and hygeine while the patient is at home. Includes references to family compliance to treatment. |
| Building relationships | Healing relationships provide emotional support, guidance, and understanding. Such relationships are built on trust, rapport, and mutual understanding of each other's roles and responsibilities. Clinicians can facilitate a healing relationship by engaging in partnership building, eliciting goals and values of the patient and family, and displaying warmth and empathy in communication. |
| Responding to emotions | Parents can experience a range of emotions, including fear, sadness, anger, anxiety, and depression. (Example: "they have a bad temper") Effective communication can respond to emotions that are apparent or anticipate emotional responses likely to develop |
| Supporting hope | Hope is essential for parents as they live with the terrifying possibility that their child might die or have significant impairments because of cancer or its treatment. Effective communication can bolster a parent's sense of hope. Includes references of hope from the provider and the parent point of view. Includes parents seeking reassurance of positive outcomes and parent referencing having hope for their child and wanting health for their child. |
| Providing validation | Many parents doubt their quality as a parent or feel a sense of guilt or shame when their child has cancer. Effective communication can validate the current experiences and concerns of the parent while also reaffirming their role in the treatment of their child. |
| Trust | Patient centered communication depends on trust between the patient/family and the medical team. Clinicians can build trust in a single encounter or overtime. Parents may implicitly trust medical providers, or may develop trust over time or as they see improvement in their child's condition. includes mentions of lack of trust or distrust. |
